# A Study of Internal Thoracic Arteriovenous Principal Perforators by Using Multi-detector Row Computed Tomography Angiography

**Published:** 2016-02-11

**Authors:** Ko Okumura, Kazunobu Hashikawa, Shunsuke Sakakibara, Hiroyuki Onishi, Hiroto Terashi

**Affiliations:** ^a^Department of Plastic and Reconstructive Surgery, Kobe University Graduate School of Medicine, Hyogo, Kobe, Japan; ^b^Department of Plastic and Reconstructive Surgery, Shinko Hospital, Hyogo, Kobe, Japan; ^c^Kobe Cardiovascular Clinic, Hyogo, Kobe, Japan

**Keywords:** internal mammary perforators, multi-detector row computed tomography angiography, anatomy, principal perforator, breast reconstruction

## Abstract

**Objective:** There are numerous reports of perforating branches from the intercostal spaces of the internal thoracic vessels. These branches have varying diameters, and a main perforating branch, the principal perforator, most often found in the second or third intercostal space. We report different results based on multi-detector row computed tomography. **Methods:** We evaluated 121 sides from 70 women scheduled for breast reconstruction with free lower abdominal skin flaps who underwent preoperative multi-detector row computed tomographic scan between June 2008 and June 2015. For primary reconstruction, we analyzed both sides, and for 1-sided secondary reconstruction, we analyzed only the unaffected side. We evaluated both early arterial phase and late venous phase 5-mm horizontal, cross-sectional, and volume-rendering images for perforation sites and internal thoracic arteriovenous perforating branches’ intercostal space thickness. We analyzed differences in thickness between the internal thoracic arteries and veins and symmetry in cases involving both sides. **Results:** Venous principal perforators nearly always perforated the same intercostal spaces as accompanying veins of arterial principal perforators (99.2%), forming arteriovenous principal perforators. We found 49 principal perforators in the first intercostal space (37.4%), 52 in the second intercostal space (39.7%), 23 in the third intercostal space (17.6%), 6 in the fourth intercostal space (4.6%), and 1 in the fifth intercostal space (0.7%). Of the 51 cases in which we studied both sides, 25 cases (49%) had principal perforators with bilateral symmetry. **Conclusions:** In contrast to findings from past reports, we found that internal thoracic arteriovenous principal perforators were often present in almost the same numbers in the first and second intercostal spaces.

There are numerous reports on vessels involved in perforating branches running from the intercostal spaces of internal thoracic arteries and veins, and they have long been the subject of anatomical studies, partially because they provide pedicles for deltopectoral skin flaps.^1^^-^10 In addition, recent reports described their usefulness as recipient blood vessels in breast reconstruction using free skin flaps.^11^^-^16 According to past studies, perforating branches from each of the intercostal spaces have different diameters; those with large diameters are referred to as principal perforators, and opinions differ widely as to their locations.^4^ However, it is often reported that principal perforators are frequently found in the second or third intercostal space.^1^^,^^3^^,^^12^^,^^17^^,^^18^

Recently, there has been significant progress in imaging-based diagnosis, and the advent of multi-detector row computed tomography (MDCT) has allowed detailed 3-dimensional imaging of artery dissection at the perforating branch level. Multiple studies report its usefulness in preoperative testing for perforator flap surgery in the plastic and reconstructive surgery field.^19^^-^22 Preoperative contrast-enhanced MDCT also allows close examination of the inferior epigastric artery before breast reconstruction with free lower abdominal skin flaps. Close examination is performed while scanning the main branches of the internal thoracic arteries and veins serving as recipient blood vessels, as well as their perforating branches. In several cases, we have observed perforating branches that could be called principal perforators for both arteries and veins in the first intercostal space, which is not said to be their usual location.

Here, we report different results from those in past studies that were obtained through close examination of recent MDCT data to determine the positions of principal perforators.

## METHODS

We examined 121 sides from 70 women scheduled for breast reconstruction with free lower abdominal skin flaps who underwent preoperative MDCT scan at our hospital between June 2008 and June 2015. The study was performed with the approval of our institutional review board, and patients provided informed consent for this. For primary reconstruction, we examined both sides, and for 1-sided secondary reconstruction, we examined only the unaffected side. We excluded cases in which there was a history of irradiation at the target site or when past surgical stress was suspected. All MDCT scans were obtained at the same facility (Kobe Cardiovascular Clinic) using a CT device (Siemens SOMATOM Definition, AS+, 64-slice, Siemens 1-11-1 Osaki, Shinagawaku, Tokyo, Japan). The same scanning conditions were set for all cases (Table 1), and scanning began after spraying the oral cavity with a nitrous acid agent. We observed both early arterial phase and late venous phase images in 5-mm slice thickness/5-mm slice interval horizontal, cross-sectional images and volume-rendering images of the chest wall, and we recorded the perforation sites and thicknesses in the intercostal spaces of the internal thoracic arteriovenous perforating branches, the differences in thickness for the internal thoracic arteries and veins, and perforating branch position symmetry in cases involving both sides.

## RESULTS

**Figure 1:**
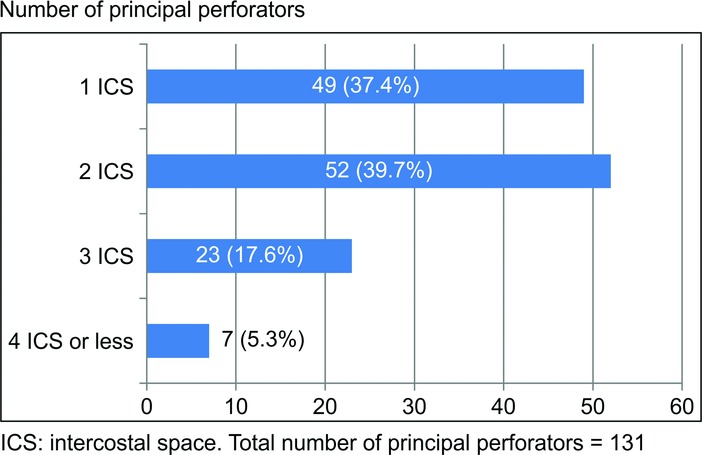
Number of principal perforators.

We found 1 to 2 blood vessels that were definitely thicker than the perforating branches in other intercostal spaces in all 121 sides in early arterial phase images, totaling 131 in number. In almost all cases, there was 1 thick perforating branch per side (113 sides; 93.4%), only a few cases with 2 perforating branches of the same thickness per side (8 sides; 6.6%), and no case with 3 or more. However, the perforating branches themselves could often not be clearly rendered in other intercostal spaces. We found 49 thickest perforating branches in the first intercostal space (37.4%), 52 in the second intercostal space (39.7%), 23 in the third intercostal space (17.6%), 6 in the fourth intercostal space (4.6%), and 1 in the fifth intercostal space (0.7%) (Fig 1). Of these, none were rendered more thickly than were the main branches of the internal thoracic arteries. In the 51 cases in which both sides were studied, there were 25 cases (49%) in which the positions of the thickest perforating branches on both sides had bilateral symmetry. In late venous phase observations, all of the thickest perforating veins, except one, were present as accompanying veins of the thickest perforating arteries and perforated the same intercostal space. The one exception perforating vein joined the perforating artery from the first intercostal space within the pectoralis major muscle as an accompanying vein, as the artery perforated the second intercostal space such that it crossed over the second costal cartilage.

## DISCUSSION

According to multiple past reports, it is now generally thought that internal thoracic arteriovenous principal perforators often perforate the second or third intercostal space.^1^^,^^3^^,^^12^^,^^17^^,^^18^ However, in the present study, almost the same number of perforators was found perforating from the first intercostal space (49 perforators; 37.4%) and from the second (52 perforators; 39.7%) intercostal space. Others were much fewer in number, with half or fewer in the third intercostal space (23 perforators; 17.6%) and the fourth intercostal space and below (7 perforators; 5.3%). These results are clearly different from those reported previously.

Examining the investigative methods in past reports, we find investigation through fresh cadaver dissection,^1^^,^^4^^-^8^,^^11^ findings during surgery,^12^^,^^13^ and, recently, investigation through CT or magnetic resonance imaging (MRI)^10^ (Table 2).

In investigations of fresh cadaver dissection, the locations of principal perforators are consistent, showing the most perforating branches in the second intercostal space. However, with respect to perforators originating from the first intercostal space, findings are discrepant; one study reported that first intercostal space perforating branches are rare,^11^ whereas another reported that the second most numerous were those originating in the first intercostal space.^4^ In addition, another study reported that second intercostal space perforating branches were usually the thickest, although those in other intercostal spaces could average 1 mm or more in outer diameter; in cases in which second intercostal space perforating branches were not thick, first or third intercostal space perforating branches were.^6^ Otherwise, arteries originating from the first intercostal space are infrequent, but veins originating from the first intercostal space are reportedly the second most common.^5^ Investigations using fresh cadaver dissection appear to be highly reliable since actual structures receive direct visual confirmation, but since living bodies with circulating blood are not involved, it is possible that the findings may differ from reality. It is also thought that investigative results may not be consistent due to variations in dissection and measurement among researchers. Finally, of the reports surveyed here, the largest number of bodies used in a study^4^ was 31; otherwise, about 10 bodies were generally used.^5^^-^7^,^^11^ A qualitative flaw in these studies is that research involving large numbers is difficult.

In studies using surgical findings, principal perforators were often observed in the second and third intercostal spaces. Since these involved actual living bodies, they are believed to be the most factual. However, previous investigations involved breast reconstruction cases during breast resection,^12^^,^^13^ so there are possible effects from surgical stress and the first intercostal space may not have been cut in all cases; therefore, accurate comparison is difficult.

**Figure 2:**
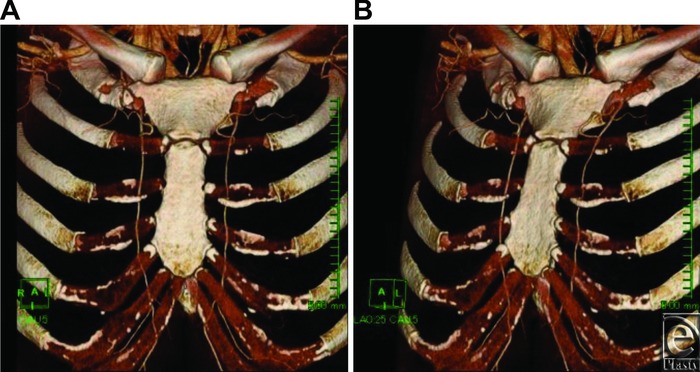
Case 1. Early arterial phase chest wall volume-rendering images: (a) frontal view; (b) oblique view. Principal perforator arteries were found in the first intercostal space on both the left and right sides. No other intercostal perforators clearly rendered.

In investigations using contrast-enhanced MDCT, direct visual observation of actual subjects is not possible, but MDCT offers the advantage of being noninvasive and reflecting the thickness of blood vessels when actual blood is flowing. When the effect of image artifacts is considered, it is not possible to evaluate differences in blood vessels of very similar diameter in contrast-enhanced MDCT images by measuring their slight image-based differences. However, in each of the present images, only the perforating branches, referred to as principal perforators, were definitely rendered thickly and the perforating branches in other intercostal spaces were rendered thinly or to such a degree as to make confirmation difficult. As there are definite differences in their blood flow volume, we do not think there are problems in using this technique for defining principal perforators (Fig 2). Since the perforating branches clearly rendered in contrast-enhanced MDCT are blood vessels with a high influx of contrast agent, indicating high actual blood flow volume, these perforating branches may be referred to as principal perforators not only for their thickness but also for their blood flow volume, which is important from a clinical perspective. Since we used a 5-mm slice thickness and 5-mm slice intervals for the horizontal cross-sections in the present MDCT images, there were no sections omitted from the slices. In preparing 3-dimensional volume-rendering images, since sharp images are created using slightly overlapping data with 1-mm slice thickness and 0.8-mm slice intervals, image reliability was high. When researching subjects as in the present study, in which perforating branches were not clearly concentrated in one intercostal space and were distributed differently depending on the case, a large number of cases increase data accuracy. That it was clearly easier than in past reports to investigate many cases, MDCT is considered to be a superior research method with higher data reliability.

In past reports studying internal thoracic artery perforating branches with MDCT and other forms of imaging, 46% were located in the second intercostal space, 33% in the first intercostal space, and 20% in the third intercostal space in a report by Kim et al,^14^ and most (56.3%) were in the second intercostal space and in each of the other intercostal spaces in the tens of percents in a report by Nakatani et al.^9^ However, these 2 reports included all perforating branches rendered, regardless of their size. Since these reports did not define the thickest branches as principal perforators and the locations were not studied, it cannot be compared with our research data. In a report by Schellekens et al,^10^ the distribution of principal perforators was recorded, and the results reported are 42% in the first intercostal space, 43% in the second intercostal space, 8% in the third intercostal space, and the rest are miniscule. These agree with our results and support our findings. However, the investigative methods were not consistent, as MDCT was used to study 48 sides in 24 cases and MRI was used to study 24 sides in 12 cases. The number of subject cases was also fewer than ours. In addition, there are no reports in which perforating veins were studied simultaneously with MDCT, as in our study.

**Figure 3:**
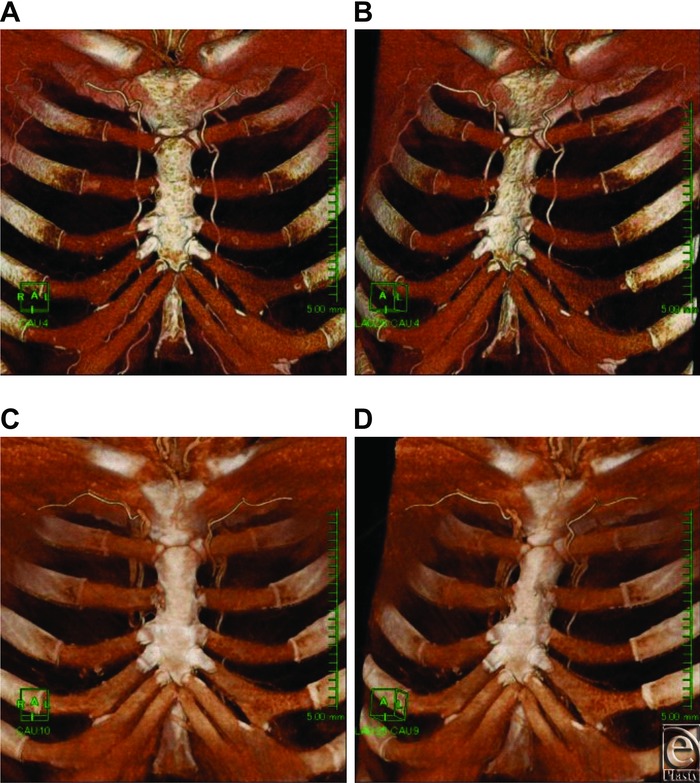
Case 2. Early arterial phase chest wall VR images: (a) frontal view; (b) oblique view. Early arterial phase and late venous phase fusion images: (c) frontal view; (d) oblique view. Principal perforators found in the first intercostal space on the right side and in the second intercostal space on the left side. Accompanying veins in the same intercostal spaces are clearly and thickly rendered in fusion images. A thin perforator is rendered in the third intercostal space on the right side.

In contrast-enhanced MDCT using the Sakakibara et al^23^ method, perforating veins were clearly rendered in the late venous phase, and it was possible to simultaneously study perforating veins (case 2, Figs 3*c* and 3*d*). When principal perforators are to be used in surgery, not only perforating arteries but also accompanying perforating veins thick enough to be called principal perforators are sought. Here, except in one case, the positions at which intercostal spaces were perforated by the thickest perforating artery and by the thickest perforating vein were not different, confirming that the artery and its accompanying vein had sufficient thickness to be principal perforators. In one case in which the perforated intercostal spaces were different, the accompanying vein perforated the intercostal space and then immediately joined the artery within the pectoralis major muscle; thus, we assumed that there would be no major problems if it were used in surgery. However, although the vein could be a principal perforator, there were significant individual differences in thickness of the perforating vein. Some veins were almost as thick as the main branches of the internal thoracic vein. On the contrary, some veins were so narrow that they caused difficulty during surgery. Thus, preoperative imaging is particularly important for perforating veins.

All of our case patients were Asians. Therefore, caution is required when discussing blood vessel dissection as in this study, as differences between races need to be considered.

The results of the present study indicate that at least among Asians, there definitely exists 1 thick principal perforator (sometimes 2) in the left and right sides among the intercostal perforating branches of the internal thoracic arteries compared with the other intercostal spaces according to past reports. This result is the same for veins and principal perforators formed from accompanying arteries and is not limited to ones with bilateral symmetry, and principal perforators are often located in the first or second intercostal space and are infrequent in the spaces below that.

Accordingly, when planning to either use internal thoracic arteriovenous perforating branches as recipient blood vessels in breast reconstruction or elevate a pedicle or free deltopectoral skin flap, the position and thickness of the principal perforators should be confirmed using proper imaging beforehand.

## Figures and Tables

**Table 1:** Multi-detector row computed tomography scanning conditions

Basic conditions	
Tube voltage	120 kV
Tube current	200 mA/rot
Helical pitch	0.8
Scanning time	˜8 s
Image reconstruction	
Slice thickness	1.0 mm
Slice intervals	0.8 mm
Reconstruction function	B30f
Contrast agent	
High-concentration contrast agent (iodine content, 350 mg/mL)	100 mL
With physiological saline	30-mL flush
Scan timing	
Bolus tracking method	
Inferior epigastric artery: Visual observation by common iliac artery	
Injection speed	3.5 mL/s
Caudocranial projection	
Reverse direction scanning during the late venous phase after scanning during the early arterial phase as above	

**Table 2:** Summary of past reports on the position of principal perforators*

	Palmer and Taylor (1986),^4^ anatomical (*n* = 40)	Munhoz et al (2004),^11^ anatomical (*n* = 22)	Munhoz et al (2004),^11^ clinical (*n* = 29)	Rosson et al (2005),^5^ anatomical (arterial) (*n* = 20)	Rosson et al (2005),^5^ anatomical (venous) (*n* = 20)
1 ICS	23%	5%	NR	10%	25%
2 ICS	57%	63%	69%	50%	40%
3 ICS	10%	27%	17%	25%	20%
4 ICS	10%	5%	4%	10%	10%
	**Saint-Cyr et al (2007),**^12^ **clinical (*n* = 27)**	**Paes et al (2011),**^7^ **anatomical (*n* = 27)**	**Schellekens et al (2011),**^8^ **anatomical (*n* = 27)**	**Halim and Alwi (2013),**^13^ **clinical (*n* = 30)**	**Schellekens et al (2013),**^10^ **CT, MRI (*n* = 72)**
1 ICS	NR	6%	15%	3%	42%
2 ICS	41%	83%	67%	63%	43%
3 ICS	53%	11%	19%	22%	8%
4 ICS	6%	0%	NR	12%	3%

*ICS indicates intercostal space; NR, not reported; CT, computed tomography; MRI, magnetic resonance imaging.
